# Management of Myelofibrosis during Treatment with Ruxolitinib: A Real-World Perspective in Case of Resistance and/or Intolerance

**DOI:** 10.3390/curroncol29070395

**Published:** 2022-07-15

**Authors:** Massimo Breccia, Francesca Palandri, Paola Guglielmelli, Giuseppe Alberto Palumbo, Alessandra Malato, Francesco Mendicino, Alessandra Ricco, Emanuela Sant’Antonio, Mario Tiribelli, Alessandra Iurlo

**Affiliations:** 1 Hematology, Department of Precision and Translational Medicine, Policlinico Umberto 1, Sapienza University, 00161 Rome, Italy; 2 Istituto di Ematologia “Seràgnoli”, IRCCS Azienda Ospedaliero-Universitaria di Bologna, 40138 Bologna, Italy; francesca.palandri@unibo.it; 3 Center of Research and Innovation of Myeloproliferative Neoplasms, AOU Careggi, University of Florence, 50134 Florence, Italy; paola.guglielmelli@unifi.it; 4 Dipartimento di Scienze Mediche, Chirurgiche e Tecnologie Avanzate “G.F. Ingrassia”, University of Catania, 95124 Catania, Italy; palumbo.gam@gmail.com; 5 UOC di Oncoematologia Ospedali Riuniti Villa Sofia-Cervello Palermo, 90146 Palermo, Italy; alessandramalato@hotmail.com; 6 Hematology Unit, Department of Hemato-Oncology, Ospedale Annunziata, 87100 Cosenza, Italy; f.mendicino@aocs.it; 7 Department of Emergency and Organ Transplantation (DETO), Hematology Section, University of Bari, 70121 Bari, Italy; alericcomd@gmail.com; 8 Department of Oncology, Division of Hematology, Azienda USL Toscana Nord Ovest, 55100 Lucca, Italy; santantonio.emanuela@hsr.it; 9 Medical Genetics, University of Siena, 53100 Siena, Italy; 10 Division of Hematology and Bone Marrow Transplantation, Department of Medical Area, University of Udine, 33100 Udine, Italy; mario.tiribelli@uniud.it; 11 Hematology Division, Foundation IRCCS Ca’ Granda Ospedale Maggiore Policlinico, 20122 Milan, Italy; alessandra.iurlo@policlinico.mi.it

**Keywords:** anemia, infection, intolerance, myelofibrosis, non-melanoma skin cancer, resistance, ruxolitinib, thrombocytopenia

## Abstract

The development and approval of ruxolitinib, the first JAK1/2 inhibitor indicated to treat myelofibrosis, has improved patient outcomes, with higher spleen and symptoms responses, improved quality of life, and overall survival. Despite this, several unmet needs remain, including the absence of resistance criteria, suboptimal response, the timing of allogeneic transplant, and the management of patients in case of intolerance. Here, we report the results of the second survey led by the “MPN Lab” collaboration, which aimed to report physicians’ perspectives on these topics. As in our first survey, physicians were selected throughout Italy, and we included those with extensive experience in treating myeloproliferative neoplasms and those with less experience representing clinical practice in the real world. The results presented here, summarized using descriptive analyses, highlight the need for a clear definition of response to ruxolitinib as well as recommendations to guide the management of ruxolitinib under specific conditions including anemia, thrombocytopenia, infections, and non-melanoma skin cancers.

## 1. Introduction

Primary myelofibrosis (MF) is a myeloproliferative neoplasm characterized by bone marrow fibrosis, hepato-splenomegaly, constitutional symptoms, and progressive anemia that can affect quality of life [1,2]. Secondary MF can also occur following myelofibrotic transformation of polycythemia vera (PV) and essential thrombocythemia (ET), termed post-PV MF and post-ET MF, respectively [2]. Patients with primary MF have a reduced overall survival (OS), with median OS ranging from 2.3 to 11.3 years after diagnosis according to the baseline International Prognostic Scoring System (IPSS) risk score [3,4].

Ruxolitinib is an oral inhibitor targeting JAK1 and JAK2. Treatment with ruxolitinib was associated with spleen response and improvement of symptoms and OS in patients with intermediate-2 risk or high-risk primary or secondary MF in the COMFORT-I and COMFORT-II phase 3 trials [5,6,7]. These findings were confirmed by the phase 3 JUMP trial, which evaluated ruxolitinib in MF patients with intermediate-1 risk or low platelet counts and those matching the eligibility criteria of the COMFORT trials [8].

Approximately 50% of patients with MF discontinue ruxolitinib due to failure or intolerance after a median follow-up of 37 months [9]. Furthermore, the survival of patients with MF after discontinuation of ruxolitinib is poor, with a reported median OS of 14 months [10]. Clonal evolution while receiving ruxolitinib or thrombocytopenia at ruxolitinib discontinuation were also associated with poor prognosis [10]. However, the optimal management of MF patients who experience failure and/or intolerance to ruxolitinib, and the best way to define resistance criteria, remain to be clarified. Hence, this project aimed to collect physicians’ perceptions on the definition of suboptimal and failure responses, the management of ruxolitinib during intolerance, and the timing and role of allogeneic transplant.

## 2. Materials and Methods

The “MPN Lab” collaboration was established in March 2018 to examine experiences, perspectives, and proposals on the management of MF and PV from 18 Italian hematology centers. Their first survey was published in 2020 and described the processes involved in diagnosing, stratification, and managing MF patients in real-world practice [11]. A second survey, consisting of 27 questions, was then developed to assess the identification of resistant and intolerant MF patients, the role of allogeneic transplant, and physicians’ perceptions on novel therapeutic strategies. The complete survey questionnaire, and responses, are provided in Supplementary Material Table S1. Similar to the first survey, the questionnaire was distributed via the internet and an external agency collected the responses. Due to the COVID-19 pandemic, participants met via an online meeting to discuss the final results. Here we present the results of the second survey summarized by descriptive statistics.

## 3. Results

As with our first survey [11], participants were selected from the overall Italian geographical region and included physicians with extensive experience in treating myeloproliferative neoplasms and those with less experience, representing a real-world scenario. Overall, 28 hematologists all across Italy completed the survey; 13 with less experience (less than 50 MF patients treated with ruxolitinib in their hospital) and 15 with higher experience (≥50 MF patients treated with ruxolitinib).

### 3.1. Identification of Resistance

**Figure 1:**
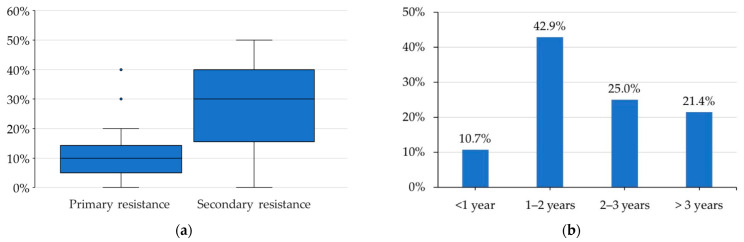
Participant responses: (**a**) Among your myelofibrosis patients who started ruxolitinib treatment, how many presented primary and secondary resistance? (**b**) Among patients who have experienced secondary resistance to ruxolitinib, after how long on average has there been a loss of response to the drug?

Initially, participants were asked to quantify primary and acquired resistance to ruxolitinib. They reported that a median of 10% (interquartile range (IQR) 5–13%) of their patients could be defined as primary resistant. Most physicians defined primary resistance as the lack of a splenic volume response ≥ 35% (SVR35%) at 6 months (54%), while others defined it as the lack of SVR50% at 6 months (29%) or the lack of SVR35% at 3 months (14%). A median of 14% (IQR 5–31%) of patients with MF and primary resistance to ruxolitinib initiated therapy at a dose < 15 mg BID regardless of platelet counts, which may explain the lack of SVR35%. Indeed, the median percentage of patients with acquired resistance was 30% (IQR 17–40%) (Figure 1a). Most physicians (43%) reported that the loss of response to ruxolitinib developed in patients after 1–2 years of therapy, while approximately 20% of patients developed resistance to ruxolitinib after a dose reduction due to the onset of adverse events (Figure 1b).

**Figure 2:**
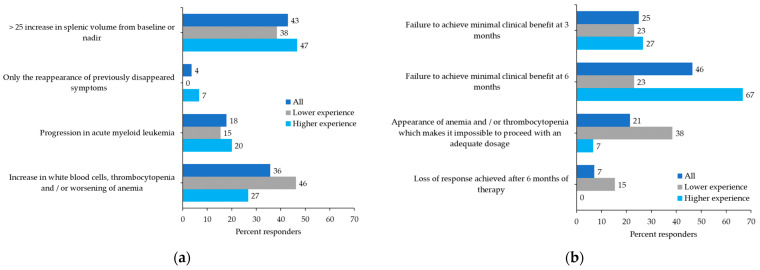
Participant preferences: (**a**) How do you define disease progression? (**b**) How do you define a suboptimal response to ruxolitinib?

Definitions of disease progression varied widely and included a >25% increase in SV from baseline or nadir (43%), an increase in white blood cell (WBC) count, thrombocytopenia and/or worsening of anemia (36%), progression in acute myeloid leukemia (18%), or the reappearance of symptoms (4%) (Figure 2a). No consensus was reached on the definition of suboptimal response, although a trend is visible among hematologists with higher experience with ruxolitinib in MF (Figure 2b). Most participants reported suboptimal response as a failure to achieve a minimal clinical benefit at 6 months (46%) or 3 months (25%), while the onset of anemia and/or thrombocytopenia that did not allow treatment to proceed with an adequate dosage of ruxolitinib was reported by 21%.

### 3.2. Treatment in Case of Resistance

**Figure 3:**
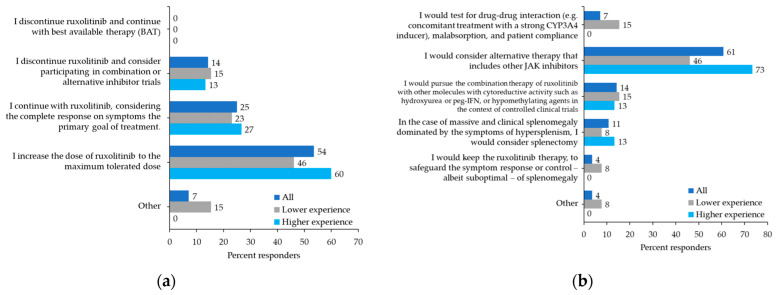
Participant preferences: (**a**) Leukocytosis/response on symptoms only: full control of systemic symptoms may be achieved in the patient receiving ruxolitinib, even in the presence of uncontrolled myeloproliferation (persistence of splenomegaly, hyperleukocytosis). If so, which therapeutic approach would you consider? (**b**) Splenomegaly due to failure to respond to adequate or maximum tolerated dose (resistance): how do you manage the problem of primary resistance splenomegaly or loss of response to ruxolitinib?

Physicians were asked about their behavior in particular situations. For MF patients receiving ruxolitinib with persistent splenomegaly and leukocytosis, 54% of physicians would increase the dose of ruxolitinib to the maximum tolerated dose, 25% would continue the same dose and consider symptoms response as the primary endpoint, and 14% would discontinue the drug and search for an alternative trial (Figure 3a). More than half of the participants (57%) would prescribe a second-line JAK2 inhibitor as soon as possible for patients with primary resistance to ruxolitinib. In comparison, 25% would anticipate switching a patient with a suboptimal response after a minimum of 3–6 months of treatment with ruxolitinib and 18% would switch a patient who developed a secondary resistance after at least 6 months of continuous ruxolitinib treatment. To manage persistent splenomegaly due to the failure to respond to an adequate or maximum tolerated dose of ruxolitinib, 61% of physicians would consider an alternative therapy including other JAK2 inhibitors. Conversely, 14% would continue with ruxolitinib associated with conventional agents, such as hydroxyurea or interferon, and 11% would consider splenectomy (Figure 3b).

### 3.3. The Role of Allogeneic Transplant

**Figure 4:**
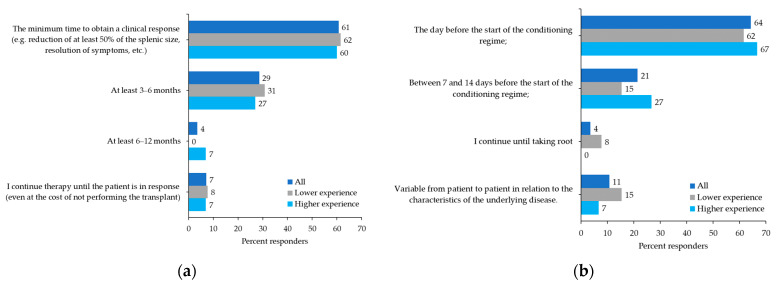
Participant preferences: (**a**) In an allogeneic transplant candidate patient, what do you think is the optimal period of JAK inhibitor therapy prior to transplantation? (**b**) In a transplant candidate patient, what is the timing of withdrawal of the JAK inhibitor adopted by your center?

The survey included specific questions regarding the role of JAK inhibitor therapy in hematopoietic stem cell transplantation (HSCT). In detail, in an MF patient with intermediate-1 IPSS risk and an available donor, 75% of physicians believed that the leading indication for HSCT was the presence of high molecular risk (HMR) mutations, whereas only 14% would consider HSCT for resistance to JAK2 inhibitors. Regarding the second question related to the optimal length of time of JAK inhibitor treatment prior to HSCT, 61% of physicians reported the absence of a predetermined period and instead considered the minimum time to obtain a clinical response, while 29% indicated a minimum period of 3–6 months of treatment (Figure 4a).

When questioned on the timing of ruxolitinib withdrawal in a transplant candidate patient before starting the conditioning regimen, 64% of participants said they would discontinue ruxolitinib the day before, 21% said between 7 and 14 days before, whereas 11% reported that this was variable between patients and related to the underlying pathology (Figure 4b). Only 4% would continue with ruxolitinib until engraftment. Regarding the use of JAK2 inhibitors after allogeneic transplantation, 54% of participants would use them for persistent minimal residual disease, 32% for overt relapse, whereas only 4% would always use them as a pre-emptive treatment strategy.

In the absence of an HLA-identical donor, more than half of the participants (54%) would use a haploidentical donor if HSCT was indicated, whereas 11% would only use a haploidentical donor in younger patients (<40 years) or in patients with high-risk disease. Most physicians (75%) would use splenectomy before transplant in case of massive splenomegaly. There was no consensus on whether splenic radiotherapy was beneficial in an HSCT candidate with MF; 46% of physicians found the procedure to be useful in patients with a contraindication to splenectomy and lack of efficacy to ruxolitinib, while 40% of physicians did not agree with this procedure.

### 3.4. Treatment in Case of Intolerance

**Figure 5:**
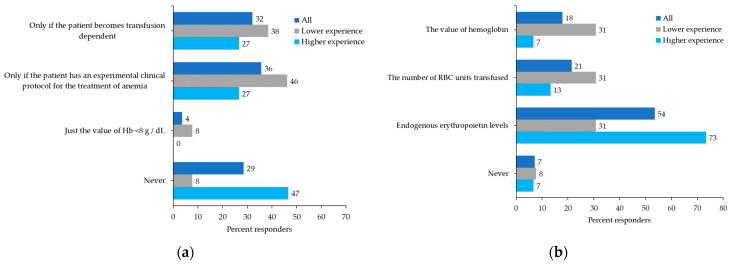
Participant preferences: (**a**) In a patient with MF receiving ruxolitinib and clinical response (CR, PR) but with anemia, when do you feel it is necessary to stop or reduce the dose of treatment? (**b**) In a patient with MF receiving ruxolitinib and clinical response (CR, PR) but developing anemia, which do you think is the best parameter for deciding to combine an erythropoietin stimulating agent (ESA)?

The survey also questioned how physicians would manage hematological side effects and infections in a patient with MF receiving ruxolitinib who has achieved a clinical response. For patients who develop anemia, 36% of physicians would interrupt or reduce the dose of ruxolitinib if other treatments for anemia were initiated, whereas 32% would only discontinue or reduce the dose in patients with transfusion dependence; 29% would never discontinue the drug, especially among the ones with more experience treating MF patients with ruxolitinib (Figure 5a). To manage anemia during ruxolitinib treatment, more than 40% of physicians would consider initiating concomitant treatment with erythropoietin. When asked which parameter indicated when to initiate an erythropoiesis-stimulating agent, most physicians (54% or 73% among the more experienced) would only consider endogenous erythropoietin levels (Figure 5b). Similarly, in the case of thrombocytopenia, 64% of physicians would only discontinue or reduce the dose of ruxolitinib in patients with hemorrhagic events during treatment. Alternative treatment or participation in a clinical trial was reported as the most appropriate management of severe thrombocytopenia by 68% of physicians.

**Figure 6:**
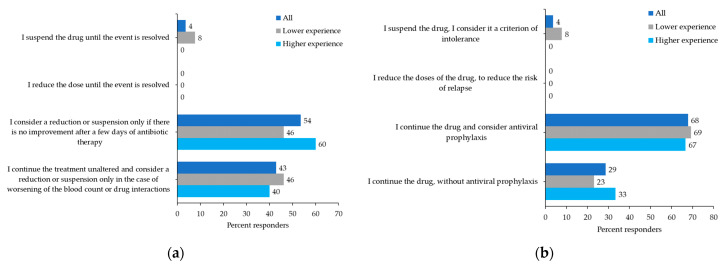

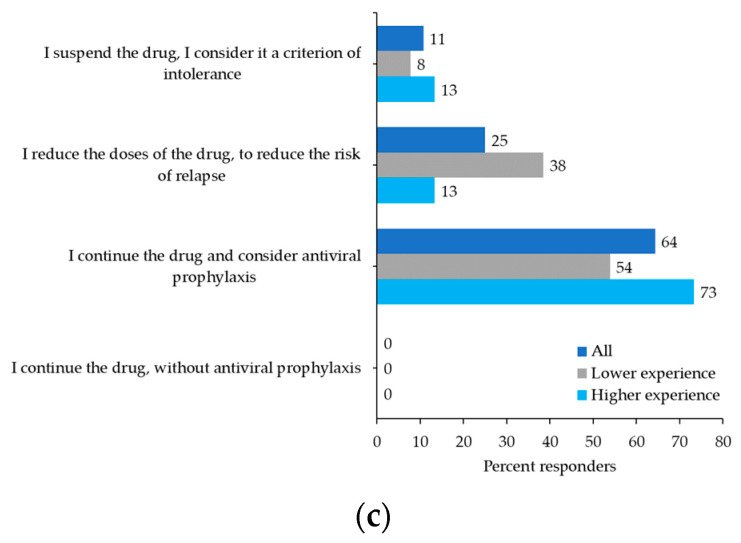
Participant preferences: (**a**) How do you manage treatment in a patient with myelofibrosis (MF) on stable doses of ruxolitinib who has an intercurrent bacterial infectious event? (**b**) How do you manage treatment in a patient with MF on stable doses of ruxolitinib who has a first episode of herpes zoster reactivation or (**c**) who has two or more episodes of herpes zoster reactivation?

For patients with dermatologic complications and the onset of non-melanoma skin cancer (NMSC), 57% of participants would only discontinue ruxolitinib in patients with recurrent NMSCs (i.e., surgical removal of at least five lesions). There was no consensus on the management of patients with a single bacterial infectious event; 54% of participants would only consider discontinuing or reducing the dose of ruxolitinib in the absence of improvement after several days of antibiotics (Figure 6a), whereas 43% would continue ruxolitinib and only consider discontinuation or dose reduction in a patient with worsening hematologic parameters. For patients with a first episode of herpes zoster reactivation, 68% of participants would continue ruxolitinib and consider antiviral prophylaxis (Figure 6b); likewise, 64% of participants would manage treatment for patients with recurrence of herpes zoster reactivation in the same way (Figure 6c).

## 4. Discussion

Most MF patients treated with ruxolitinib achieve at least some degree of spleen size reduction and rapid symptoms response. However, approximately half the patients, including those with an optimal response, may relapse after a median of 3 years of treatment. Moreover, in the long-term follow-up of the COMFORT II study, the probability of maintaining a spleen response after 5 years of treatment was 0.48 (95% CI, 0.35–0.60) for the patients who achieved a ≥35% spleen reduction on ruxolitinib [6]. A variable range of discontinuation has also been reported in clinical practice [12,13], while approximately 40% of patients discontinued ruxolitinib after a median of 3 years in a retrospective Italian study [9]. Our survey results align with the published literature, with 40% of patients resistant to ruxolitinib due to primary resistance (10%) or acquired resistance (30%).

Until now, there has been no consensus definition of either ruxolitinib failure or suboptimal response. Suboptimal response has been recently indicated among eligibility criteria in sponsored investigational trials investigating new potential ruxolitinib-based combinations, although its definition varies between protocols. Primary resistance to ruxolitinib is rare and defined as the absence of any benefit in the spleen and symptoms response. Secondary or acquired resistance has been defined as the loss of response (≥50% increase in spleen length from best response) or suboptimal response (<25% reduction in spleen length after at least 3 months of optimally dosed JAK2 inhibitor treatment) to ruxolitinib [14], while other groups also include intolerance and disease progression [15]. Our survey has highlighted the heterogeneity used to define treatment failure, suboptimal response, progression, and the need for a consensus on these definitions. Most of the physicians in this study considered treatment failure as the lack of SVR35% at 6 months, a suboptimal response as the lack of a minimal clinical benefit within 6 months, and progression as a >25% increase in SV from baseline or nadir or an increased WBC count together with decreased platelet counts and worsening of anemia. More recently, a prognostic model has been proposed to predict survival after 6 months of ruxolitinib in patients with MF [16].

In MF patients with primary resistance, increasing ruxolitinib to the maximum tolerated dose has been suggested, and our survey results agree with this strategy. Multivariate logistic regression models from a cooperative Italian study of 408 MF patients identified five baseline variables that reduced the probability of spleen response, including a higher disease burden (high IPSS risk score and spleen > 10 cm) and time interval from diagnosis to ruxolitinib start of >2 years [17]; therefore, the maximum tolerated dose should be used in patients with these characteristics. In patients with acquired resistance, second-line treatment with another JAK2 inhibitor was supported by most participants in our survey. Second-line JAK inhibitors, including fedratinib, momelotinib, and pacritinib, can induce clinical responses in the post-ruxolitinib setting and some evidence suggests they may be more efficacious than conventional treatments [13,15].

Currently, HSCT is the only curative option for MF, although the high rate of transplant-related morbidity and mortality should be considered [18]. As most MF patients fall within the fragile and older category with a median age of 66 years reported at the initial diagnosis [19], candidates for HSCT should be selected appropriately. When considering the situation of an intermediate-1 risk MF patient with an HLA-identical donor, most physicians surveyed here reported that the strongest indication for HSCT was the presence of HMR mutations, which is in line with consensus-based recommendations produced by the European LeukemiaNet and European Blood and Marrow Transplantation Group [20].

The clinical-molecular MF transplant scoring system (MTSS) [21] predicts post-transplantation outcomes for MF patients based on clinical, molecular, and transplant-specific information. It appears to be the most appropriate tool to select patients for transplantation. The MTSS identified age ≥ 57 years, Karnofsky performance status < 90%, platelet count < 150 × 10^9^/L, leukocyte count > 25 × 10^9^/L before transplantation, HLA-mismatched unrelated donor, ASXL1 mutation, and non-CALR/MPL driver mutation genotype as independent predictors of outcome post-transplantation. However, the optimal timing for transplantation in MF patients treated with ruxolitinib remains an open question, as highlighted by the lack of consensus reported here. Approximately 50% of patients discontinue ruxolitinib within 3–5 years [9] and the advent of new agents and combination trials with ruxolitinib as a backbone complicate treatment choice. It should also be considered that not all available JAK2 inhibitors modify disease pathogenesis and that, as indicated in the literature, an enlarged spleen may reduce engraftment and increase the risk of non-relapse mortality [22].

The optimal timing for ruxolitinib discontinuation before HSCT is not well defined. However, several retrospective studies have demonstrated a better prognosis in MF patients responsive to pretreatment with ruxolitinib before HSCT, with a significantly lower risk of relapse and faster engraftment than in patients without ruxolitinib pretreatment [23,24]. In our survey, more than 60% of physicians stated that they would discontinue ruxolitinib the day before starting the conditioning regime for HSCT. Similarly, adverse events were more common when ruxolitinib was abruptly discontinued ≥6 days before the conditioning therapy [24,25]. For this reason, ruxolitinib as a part of the conditioning regimen or until the engraftment may improve post-HSCT outcomes and is well tolerated [26].

When considering the donor type, more than half of the physicians surveyed reported that, in the absence of an HLA-identical donor, they would use a haploidentical donor if the patient was indicated for transplantation, whereas 11% of physicians would only use a haploidentical donor in younger patients (<40 years) or in those with high-risk disease. Notably, increasing evidence has been reported in favor of haploidentical donors, with similar results to HLA-matched donors [22,27].

The management of splenomegaly before transplant reached a consensus in our survey, with more than 70% of participants reporting that they would use splenectomy. However, the role of splenectomy is debatable. On the one hand, splenectomy may have a disease-modifying effect considering that additional molecular and cytogenetical abnormalities may be present in the spleen compared with bone marrow; it may also reduce the risk of graft failure and relapse-risk mortality. Conversely, splenectomy is associated with increased mortality and morbidity, with possible thrombotic events and infections post-surgery [22,28,29].

Only a small number of physicians in our survey would use a pre-emptive ruxolitinib strategy after allogeneic transplantation. In contrast, more than half would use ruxolitinib in patients with persistent or reappearing residual disease. Notably, in a small pilot study, all four patients who received ruxolitinib pre- and post-transplant achieved complete remission, with long-term survival and no acute graft-versus-host disease [30]. Conversely, complete remission was achieved by only two of nine patients who received ruxolitinib in the pre-transplant period, and remission was not achieved in three patients who never received ruxolitinib. Post-transplant ruxolitinib was also reported to be safe and feasible in a separate study of four patients, with none experiencing fungal infections or EBV reactivation and all alive and in complete remission at a median post-transplant survival of 9.4 months [31].

We also surveyed physicians’ responses to hematological toxicity in MF patients with clinical response to ruxolitinib. For patients with anemia, only 29% of physicians would continue ruxolitinib at the same dose. In comparison, more than 70% would discontinue or reduce the dose if the patient became transfusion-dependent or started another treatment for anemia. However, the best parameter for deciding when to combine ruxolitinib with an erythropoietin-stimulating agent was unclear, although around half of the participants felt that using endogenous erythropoietin levels was the correct approach. Notably, erythropoietin-stimulating agents were efficacious in improving anemia in 59 anemic MF patients treated with ruxolitinib, with an anemia response rate of 54% and minor improvements in Hb levels in an additional 15% of patients, and no thrombotic events reported [32].

In our survey, around two-thirds of the physicians would discontinue or reduce the dose of ruxolitinib in patients who develop thrombocytopenia, but only if hemorrhagic events occur. Interestingly, more than half of the physicians would stop ruxolitinib in patients with recurrent NMSCs, while most would reduce the dose or suspend ruxolitinib in patients with a worsening bacterial infection. So far, there is no published consensus on ruxolitinib management during infectious episodes or the occurrence of second neoplasia.

## 5. Conclusions

In conclusion, the results of our survey have identified a real-life perspective of ruxolitinib in patients with MF and highlight the need for guidelines to aid the identification of resistance, the optimal timing for HSCT, and the management of patients with specific intolerance.

## Data Availability

The data presented in this study are available in this article.

## Supplementary Materials

The following supporting information can be downloaded at: https://www.mdpi.com/article/10.3390/curroncol29070395/s1, Table S1: Questionnaire and related answers.
