# Supplementary material for: Management of Myelofibrosis during Treatment with Ruxolitinib: A Real-World Perspective in Case of Resistance and/or Intolerance
Source: Curr Oncol. 2022 Jul 15;29(7):4970–80. doi: 10.3390/curroncol29070395 (PMC9325304; doi:10.3390/curroncol29070395)
Supplement: Supplementary file 1 [file curroncol-29-00395-s001.zip › curroncol-1754215-supplementary.pdf]

Current Oncology

MDPI

Table S1. Questionnaire and related answers.

POSSIBLE THERAPIES/FUTURE DEVELOPMENTS IN CASE OF FAILURE/ SUB-OPTIMAL RESPONSE	All (n=28)	Lower experience (<50 ruxolitinib pts) (n=13)	Higher experience (≥ 50 ruxolitinib pts) (n=15)
1. Management of anemia during treatment *Erythropoietin, androgens, prednisone, danazol have been used to treat MF-related anemia. In this regard, your attitude in clinical practice, in addition to supportive therapy, what do you foresee?*			
Reduce the dose or stop treatment with ruxolitinib based on the severity of the anemia. Treatment with a lower dose can be resumed after recovery of the complete blood count.	25.0%	38%	13%
Consider an alternative treatment modality, including participation in clinical trials in transfusion-dependent anemia.	14.3%	15%	13%
Consider splenectomy especially if symptoms of hypersplenism are present and if the patient is a candidate for surgery.	3.6%	0%	7%
Initiating concomitant treatment with erythropoietin may be useful and practicable, although its efficacy in addition to ruxolitinib therapy is yet to be defined.	42.9%	31%	53%
Starting concomitant treatment with danazol, as demonstrated by Study NCT01732445, can be useful and practicable in counteracting anemia.	10.7%	8%	13%
Other	3.6%	8%	0%
2. Management of thrombocytopenia during treatment: *What is your attitude in case of grade 3 thrombocytopenia?*			
I discontinue ruxolitinib and start the patient on supportive therapy only	0.0%	0%	0%
I discontinue ruxolitinib and start steroid and / or danazol treatment; if the platelet count increases to permissive levels, I reintroduce ruxolitinib	28.6%	15%	40%
If a minimum effective dose of ruxolitinib cannot be maintained, I consider an alternative treatment modality, including participation in clinical trials.	67.9%	85%	53%
I consider splenectomy, especially if symptoms of hypersplenism are present, if the patient is a candidate for surgery.	0.0%	0%	0%
Other	3.6%	0%	7%
3. Splenomegaly due to failure to respond to adequate or maximum tolerated dose (resistance): *As demonstrated by the long-term follow up of the COMFORT studies, about 50% of patients lose response to ruxolitinib in the first 5 years of treatment, suggesting the acquisition of resistance by the MPN clone. How do you manage the problem of primary resistance splenomegaly or loss of response to ruxolitinib?*			
I would test for drug-drug interaction (e.g. concomitant treatment with a strong CYP3A4 inducer), malabsorption, and patient compliance.	7.1%	15%	0%
I would consider alternative therapy that includes other JAK inhibitors.	60.7%	46%	73%
I would pursue the combination therapy of ruxolitinib with other molecules with cytoreductive activity such as hydroxyurea or peg-IFN, or hypomethylating agents in the context of controlled clinical trials.	14.3%	15%	13%
In the case of massive and clinical splenomegaly dominated by the symptoms of hypersplenism, I would consider splenectomy.	10.7%	8%	13%
I would keep the ruxolitinib therapy, to safeguard the symptom response or control – albeit suboptimal – of splenomegaly.	3.6%	8%	0%
Other	3.6%	8%	0%

4. Leukocytosis / response on symptoms only: *Full control of systemic symptoms may be achieved in the patient receiving ruxolitinib, even in the presence of uncontrolled myeloproliferation (persistence of splenomegaly, hyperleukocytosis). If so, which therapeutic approach would you consider?*			
I discontinue ruxolitinib and continue with best available therapy (BAT)	0.0%	0%	0%
I discontinue ruxolitinib and consider participating in combination or alternative inhibitor trials	14.3%	15%	13%
I continue with ruxolitinib, considering the complete response on symptoms the primary goal of treatment.	25.0%	23%	27%
I increase the dose of ruxolitinib to the maximum tolerated dose	53.6%	46%	60%
Other	7.1%	15%	0%
**ROLE OF ALLOGENIC TRANSPLANT**			
5. In a patient with intermediate-1 risk MF and HLA-identical donor (family member or MUD 10/10), which of the following do you think is the strongest indication to perform an allogeneic transplant?			
Age <60 years	7.1%	15%	0%
Blasts> 1%	0.0%	0%	0%
Presence of high molecular risk mutations (HMR)	75.0%	69%	80%
Resistance to inhibitory JAK	14.3%	15%	13%
Thrombocytopenia <100,000/mm^3	3.6%	0%	7%
6. In an allogeneic transplant candidate patient, what do you think is the optimal period of JAK inhibitor therapy prior to transplantation?			
The minimum time to obtain a clinical response (e.g. reduction of at least 50% of the splenic size, resolution of symptoms, etc.)	60.7%	62%	60%
At least 3–6 months	28.6%	31%	27%
At least 6–12 months	3.6%	0%	7%
I continue therapy until the patient is in response (even at the cost of not performing the transplant)	7.1%	8%	7%
7. In a transplant candidate patient, what is the timing of withdrawal of the Jak inhibitor adopted by your center?			
The day before the start of the conditioning regime;	64.3%	62%	67%
Between 7 and 14 days before the start of the conditioning regime;	21.4%	15%	27%
I continue until taking root	3.6%	8%	0%
Variable from patient to patient in relation to the characteristics of the underlying disease.	10.7%	15%	7%
8. When do you consider using JAK inhibitors after allogeneic transplantation?			
In case of persistence of minimal residual disease (molecular, chimerism, etc.)	53.6%	62%	47%
In case of overt relapse	32.1%	31%	33%
Always (pre-emptive strategy)	3.6%	0%	7%
Never	10.7%	8%	13%
9. In the absence of an HLA-identical donor (familial or MUD 10/10), when would you use a haploidentical donor?			
Only in very young patients (e.g. <40–50 years)	10.7%	15%	7%
Only in high-risk disease	10.7%	8%	13%
Only in case of progression on JAK inhibitor therapy	25.0%	31%	20%
Always, if there is an indication for transplantation	53.6%	46%	60%
Never	0.0%	0%	0%
10. When would you use splenectomy in a transplant patient?			
In case of massive splenomegaly (eg.> 15–20 cm from the costal arch)	75.0%	69%	80%

In case of high transfusion needs	3.6%	0%	7%
In case of severe thrombocytopenia	3.6%	0%	7%
Never	17.9%	31%	7%
11. Do you find the splenic radiotherapy procedure useful in an allogeneic transplant candidate patient?			
Yes, always	10.7%	8%	13%
Yes, only in case of contraindication to splenectomy surgery and ineffectiveness of treatment with JAK inhibitor	46.4%	38%	53%
Yes, but only in case of platelet count> 100,000 mmc	3.6%	8%	0%
Never	39.3%	46%	33%
**INTOLERANCE CRITERIA**			
12. In a patient with MF receiving ruxolitinib and clinical response (CR, PR) but with anemia, when you feel it is necessary to stop or reduce the dose of treatment:			
Only if the patient becomes transfusion dependent	32.1%	38%	27%
Only if the patient has an experimental clinical protocol for the treatment of anemia	35.7%	46%	27%
Just the value of Hb <8 g / dL	3.6%	8%	0%
Never	28.6%	8%	47%
13. In a patient with MF receiving ruxolitinib and clinical response (CR, PR) but developing anemia, which do you think is the best parameter for deciding to combine an erythropoietin stimulating agent (ESA):			
The value of hemoglobin	17.9%	31%	7%
The number of RBC units transfused	21.4%	31%	13%
Endogenous erythropoietin levels	53.6%	31%	73%
Never	7.1%	8%	7%
14. In a patient with MF receiving ruxolitinib and clinical response (CR, PR) but developing thrombocytopenia after treatment, when you feel it is necessary to stop or reduce the dose of treatment:			
Only if the patient becomes transfusion dependent	17.9%	23%	13%
Only if the patient is in an experimental clinical protocol for patients with thrombocytopenia	10.7%	8%	13%
Only if the patient has repeated minor bleeding episodes or a major bleeding episode	64.3%	62%	67%
Never	7.1%	8%	7%
15. In a patient with MF receiving ruxolitinib and clinical response (CR, PR) but with non-melanoma skin neoplasms (e.g. basaliomas), when you feel it is necessary to stop treatment:			
Only if the patient has removed at least 5 skin lesions	57.1%	54%	60%
Only if the patient has removed at least 10 skin lesions	14.3%	23%	7%
Only if the patient needs skin transplant	3.6%	8%	0%
Never	25.0%	15%	33%
16. In a patient with MF on stable doses of ruxolitinib who has an intercurrent bacterial infectious event, how do you manage treatment:			
I suspend the drug until the event is resolved	3.6%	8%	0%
I reduce the dose until the event is resolved	0.0%	0%	0%
I consider a reduction or suspension only if there is no improvement after a few days of antibiotic therapy	53.6%	46%	60%
I continue the treatment unaltered and consider a reduction or suspension only in the case of worsening of the blood count or drug interactions	42.9%	46%	40%

17. In a patient with MF on stable doses of ruxolitinib who has the first episode of Herpes Zoster reactivation, how do you manage treatment:			
I suspend the drug, I consider it a criterion of intolerance	3.6%	8%	0%
I reduce the doses of the drug, to reduce the risk of relapse	0.0%	0%	0%
I continue the drug and consider antiviral prophylaxis	67.9%	69%	67%
I continue the drug, without antiviral prophylaxis	28.6%	23%	33%
18. In a patient with MF on stable doses of ruxolitinib who has two or more episodes of Herpes Zoster reactivation, how do you manage treatment:			
I suspend the drug, I consider it a criterion of intolerance	10.7%	8%	13%
I reduce the doses of the drug, to reduce the risk of relapse	25.0%	38%	13%
I continue the drug and consider antiviral prophylaxis	64.3%	54%	73%
I continue the drug, without antiviral prophylaxis	0.0%	0%	0%
**RESISTANCE CRITERIA**			
19. Among your myelofibrosis patients who started ruxolitinib treatment, how many presented:			

All	Min	Q1	Median	Q3	Max
Primary resistance: (n)	0	2,75	5	6,5	30
Primary resistance: (%)	0%	5%	10%	13%	40%
Secondary resistance: (n)	0	5	16,5	30	40
Secondary resistance: (%)	0%	17%	30%	40%	50%

Lower exp.	Min	Q1	Median	Q3	Max
Primary resistance: (n)	0	2	3	5	8
Primary resistance: (%)	0%	5%	10%	12%	40%
Secondary resistance: (n)	0	3	5	10	20
Secondary resistance: (%)	0%	10%	17%	40%	50%

Higher exp.	Min	Q1	Median	Q3	Max
Primary resistance: (n)	1	5	5	14	30
Primary resistance: (%)	1%	7%	10%	13%	30%
Secondary resistance: (n)	1	22.5	30	40	60
Secondary resistance: (%)	1%	30%	40%	48%	50%

20. How do you assess any primary resistance to ruxolitinib and at what time point?			
Failure to achieve> 35% reduction in splenic size at 3 months	14.3%	8%	20%
Failure to achieve> 35% reduction in splenic size at 6 months	53.6%	46%	60%
Failure to achieve> 50% reduction in splenic size at 3 months	3.6%	8%	0%
Failure to achieve> 50% reduction in splenic size at 6 months	28.6%	38%	20%
21. In your experience, how many patients with myelofibrosis and primary resistance to ruxolitinib have initiated such therapy at a dose of less than 15 mg BID?			

All	Min	Q1	Median	Q3	Max
(n)	0	1	3	6,5	50
(%)	0%	5%	14%	31%	90%

Lower exp.	Min	Q1	Median	Q3	Max
(n)	0	0	2	4	30
(%)	0%	0%	9%	30%	60%

Higher exp.	Min	Q1	Median	Q3	Max
(n)	0	2.5	5	15	50
(%)	0%	10%	25%	33%	90%

22. How do you define disease progression?			
> 25% increase in splenic volume from baseline or nadir	42.9%	38%	47%
Only the reappearance of previously disappeared symptoms	3.6%	0%	7%
Progression in acute myeloid leukemia	17.9%	15%	20%
Increase in white blood cells, thrombocytopenia and / or worsening of anemia	35.7%	46%	27%
23. How do you define a suboptimal response to ruxolitinib?			
Failure to achieve minimal clinical benefit at 3 months	25.0%	23%	27%
Failure to achieve minimal clinical benefit at 6 months	46.4%	23%	67%
Appearance of anemia and / or thrombocytopenia which makes it impossible to proceed with an adequate dosage	21.4%	38%	7%
Loss of response achieved after 6 months of therapy	7.1%	15%	0%
24. In your experience, among patients who have experienced secondary resistance to ruxolitinib, how long on average has there been a loss of response to the drug?			
<1 year	10.7%	8%	13%
1–2 years	42.9%	38%	47%
2–3 years	25.0%	23%	27%
> 3 years	21.4%	31%	13%
25. In your experience, in how many patients with myelofibrosis, a pattern of secondary resistance to ruxolitinib arose following a dose reduction of the drug due to toxicity?			

All	Min	Q1	Mediana	Q3	Max
(n)	0	1	8,5	11,25	70
(%)	0%	3%	20%	50%	90%

Lower exp.	Min	Q1	Mediana	Q3	Max
(n)	0	1	4	9	15
(%)	0%	4%	16%	50%	90%

Higher exp.	Min	Q1	Mediana	Q3	Max
(n)	0	1	10	25	70
(%)	0%	5%	20%	50%	60%

26. With the option of prescribing another JAK2 inhibitor, which patient would you apply to a second line and when:			
The patient in primary resistance as soon as possible	57.1%	54%	60%
The patient in secondary resistance after at least 6 months of ruxolitinib therapy	17.9%	23%	13%
The patient in suboptimal response after at least 3–6 months, regardless of future therapeutic strategy	25.0%	23%	27%
The patient who is a candidate for transplant but who has not reached an "optimal" response	0.0%	0%	0%

27. In a patient who has shown resistance (primary or secondary) what is your behavior: [rank from 1 to 3 (most important)]			
I enroll in a clinical trial (if applicable) in my center or in a referral center	2.96	2.92	3.00
I proceed with the splenectomy if the splenic encumbrance is the greatest discomfort for the patient	1.43	1.38	1.47
Other	1.61	1.77	1.47
